# Vitamin D Regulates the Expressions of AQP-1 and AQP-4 in Mice Kidneys

**DOI:** 10.1155/2019/3027036

**Published:** 2019-01-27

**Authors:** Yu Fu, Jiajun Zhu, Yalin Zhang, Zuwang Liu, Han Su, Juan Kong

**Affiliations:** ^1^Department of Clinical Nutrition, Shengjing Hospital, China Medical University, No. 36 Sanhao St., Heping District, Shenyang, China; ^2^Department of Orthopedics, The Fourth Affiliated Hospital, China Medical University, No. 4 Chongshan Dong Road, Huanggu District, Shenyang, China

## Abstract

**Aim:**

Vitamin D plays an important role in water and salt homeostasis. The aim of our study was to investigate the underlying relationship of Vitamin D and Aquaporins (AQP).

**Methods:**

The behaviors of 1*α* (OH)-ase knockout mice and wild type mice were observed before analysis. The ICR mice were treated with vehicle or paricalcitol, a vitamin D analogue, followed by animals receiving a standard diet and free access to drinking water either with aliskiren (renin blocker; 37.5 mg aliskiren in 100 ml water), or telmisartan (a angiotensin II type I receptor blocker; 40 mg telmisartan in 100 ml water) a week before study. The expressions of AQP-1, AQP-4, and renin in mice kidneys were detected by western bolting, immunohistochemistry, and immunofluorescence.

**Results:**

Diuresis and polydipsia were observed in 1*α* (OH)-ase knockout mice, and a decreased water intake and urine output in ICR mice was observed after paricalcitol treatment. Compared with wild type, the AQP-1 expressions were increased in renal papilla and AQP-4 expressions were decreased in renal proximal tubule of 1*α*(OH) ase knockout mice. In addition, AQP-1 was decreased in renal papilla and AQP-4 expressions were increased in proximal tubule by suppressing renin activity or supplement of Vitamin D analogue. After injecting renin into the lateral ventricle of the 1*α*(OH)ase knockout mice, the renin expression level was decreased in the kidney, followed by the decrease of AQP-1 in renal papilla and increase of AQP-4 in proximal tubule.

**Conclusions:**

Overall, Vitamin D and renin inhibitors have synergistic effects in regulating water channels in mice kidneys.

## 1. Background

Vitamin D, a fat-soluble steroid hormone, plays an important role in regulating a number of biological functions [1]. The Vitamin D precursors, 7-dehydrocholesterol, are formed by ultraviolet irradiation in the skin and transported to liver. Hydroxy Vitamin D_3_, 25-hydroxyvitamin D_3_, is synthesized from 7-dehydrocholesterol by 25-cholcalciferol hydroxylase in the liver and is transported to kidney. In the kidney, 1,25-dihydroxyvitamin D_3_ is produced by the action of 1*α*-hydroxylase. 1,25-dihydroxyvitamin D_3_, the activity form of Vitamin D3, as an agonist of nuclear receptor super-family dihydroxyvitamin binds to Vitamin D receptor (VDR), which is a member of nuclear receptor super family and regulate gene expression by heterodimerizing with other members and translocating to nucleus and binding to nuclear response element [2]. The role of Vitamin D_3_ in calcium and phosphate metabolism [3], blood sugar control [4], lipid metabolism, salt excretion, and urine concentration is well established. In our previous studies, we have demonstrated that VDR knockout mice cause polyuria and anadipsia [5].

Aquaporins (AQPs), also known as water channel proteins, are a family of 10 AQPs involved in the regulation of water transport, which exist in various organs [6]. AQPs can be grouped in two groups based on the permeability of anions to their AQPs. The first group of AQPs (AQP-0, AQP-1, AQP-2, AQP-4, AQP-5, and AQP-8) only permit water transport [7]. The other group represented by AQP-3, AQP-7, AQP-9, and AQP-10 serve as water but also other molecule transporters such as glycerin and other small anions [8]. AQP-1, AQP-2, AQP-3, AQP-4, and AQP-6 are expressed in kidney regulating water transport and thereby water intake and urine formation [9, 10]. AQP-1, defined as CHIP-27 in red blood cells for the first time, plays an important role in urine concentration in proximal tubules, descending thin limbs and renal vasa recta and keeping water balance in multiple water-permeable epithelial cells [11]. AQP-1 gene knocked out animals are normal phenotypes besides retaining the ability of concentrating urine. Water permeability was 8.5 folds lower in AQP-1 knockout mice than that of wild-type mice [12]. Similar to AQP-1 knockout mice, AQP-4 knockout mice have no defects in growth and survival but have reduced urine concentration capacity [13], which are also observed in VDR knockout mice [5]. This suggests that the underlying link may exist between AQPs and Vitamin D/VDR in urinary formation. This study attempts to explore the relationship between AQPs and Vitamin D/VDR mediated urinary production.

## 2. Materials and Methods

### 2.1. Animals and Design

The ICR wild-type mice purchased by Animal Center of China Medical University were used as experimental subjects, which is also the background mice of 1-alpha-hydroxylase (1*α*(OH)ase) knockout mice with deletion of CYP27B1 gene. The mice were housed in a 12 hour light/dark cycle with free access to food and water, except where otherwise indicated. ICR wild-type mice were randomly divided into 6 groups (males, 6-8weeks old, n= 8 per group). The mice of con group were treated with vehicle (4 times a week, ip), and mice of pari group were treated by paricalcitol (Paricalcitol, analogues of Vitamin D, 400 ng/kg,4 times a week, ip). The mice of water group were fed with water. Mice of telmisartan or aliskren group were fed with Telmisartan (Telmisartan, angiotensin II type I receptor blocker, 40 mg /100ml) or aliskiren (Aliskiren, renin blocker, 37.5mg /100ml) in drinking water. The mice in pari+aliskiren group were injected by paricalcitol and fed with 37.5% aliskiren water. The 1*α*(OH)ase knockout mice were divided into ko group and ko+renin group (males, 6-8weeks old, n=5-8 per group), in which renin (1*μ*l, 0.2 × 10^3^ *μ*g/*μ*l) was injected into the lateral ventricle via stereotaxic instrument. The behaviors of the mice were observed before sacrificed. All animal procedures were carried out to minimize suffering in accordance with the guidelines established by the Animal Experimental Committee.

### 2.2. Western Blot

The renal proteins were prepared using Laemmli buffer and separated by SDS-PAGE and then transferred by PVDF membranes. The membranes were blocked with 1% BSA and incubated sequentially with primary antibodies (1:1000 dilution) and secondary antibodies (1:2000 dilution). *β*-actin was used as the internal control. Western blotting was carried out using antibodies against AQP-1(sc-25287, Santa Cruz Biotechnology, USA), AQP-4 (sc-20812, Santa Cruz Biotechnology, USA), Renin (sc-22671, Santa Cruz Biotechnology, USA), and *β*-actin (sc-47778, Santa Cruz Biotechnology, USA).

### 2.3. Immunohistochemistry and Immunofluorescence

The renal tissues were fixed with 4% paraformaldehyde made in PBS (pH 7.2), embedded in paraffin, and cutted into 5 *μ*m sections. The sections were incubated with the primary antibodies of AQP-1, AQP-4 and renin, then detected after secondary antibodies labelled fluorescence or HRP. Images were acquired by fluorescence microscopy. The representative images were repeated at least twice from different sections.

### 2.4. Statistical Analysis

The data were presented as mean±SD. All data was analyzed using t-test or one-way analysis of variance (ANOVA).* P*< 0.05 was considered statistically significance.

## 3. Results

### 3.1. Regulatory Effect of Vitamin D on Water Intake and Urine Volume in Mice

**Figure 1:**
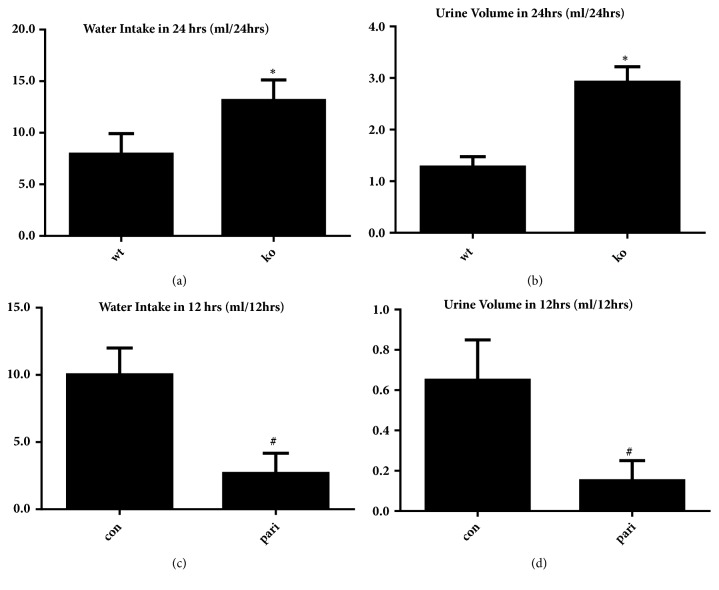
Basic situation of water intake and urine volume. The mice were put into metabolic cage 24 hrs, free accesses to water and food. (a, b) The water intake and urine volume in 1*α*(OH)ase knockout mice and wild type mice. (c, d) The water intake and urine volume of the mice treated with paricalcitol or vehicle. *∗* Compared with wt group,* P*<0.05. # Compared with con group,* P*<0.05.

Compared with wt group, the water intake and urine output of mice in ko group increased within 24 hours, and the volume of drinking water and urine was significantly decreased in pari group (Figure 1), suggesting that the Vitamin D/VDR system plays a role in regulation of urine volume and water intake.

### 3.2. Vitamin D Downregulated the Expression of AQP-1 in Renal Papilla

**Figure 2:**
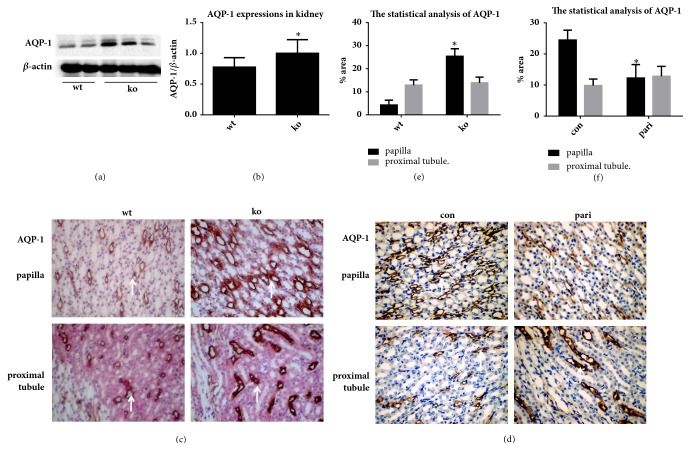
The regulation of Vitamin D on AQP-1 expressions in kidney. (a, c) The AQP-1 expressions in wild type and 1*α*(OH)ase knockout mice by western blot and immunohistochemical staining. (b) The statistic analysis of (a) by Image J. (d) The AQP-1 expressions in con group and pari group mice by immunohistochemical staining (400-fold magnifications). (e, f) The statistic analysis of (c) and (d) by Image J. Positive expression was indicated by arrows. *∗* Compared with wt group,* P*<0.05. # Compared with con group,* P*<0.05.

Compared with wild type mice, the expressions of AQP-1 increased significantly in the kidneys of 1*α*(OH)ase knockout mice (Figure 2(a)).

Although there were no differences in the proximal tubules, the expressions of AQP-1 in the renal papilla of the 1*α*(OH)ase knockout mice were significantly increased compared with the wild type mice (Figure 2(c)).

Although the expressionss of AQP-1 in the renal papillary area of pari group were significantly lower than that of the control group, there was no significant change in the proximal tubules of the two groups as shown in Figures 2(d) and 2(f), indicating that Vitamin D signaling pathway downregulated the expressions of AQP-1 in the papillary area rather than renal tubule area in mice.

### 3.3. Vitamin D and Inhibiting Renin Activity Have Synergistic Effects on Downregulation of AQP-1 Expression

Telmisartan was used as an angiotensin II type I receptor blocker to block RAS pathway and aliskiren as renin activity inhibitor to determine whether the effect of Vitamin D on AQP-1 in papillary area was through RAS system or renin activity.

**Figure 3:**
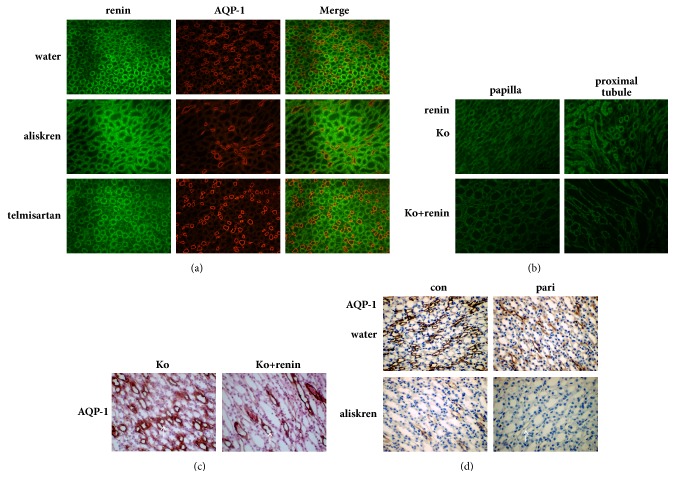
The synergistic effects of Vitamin D and inhibiting renin activity on downregulation of AQP-1 expression. (a)The coexpressions of renin and AQP-1 in renal papilla were detected by immunofluorescence staining. (b) Renin expressions in kidney were detected by immunofluorescence staining. (c, d) AQP-1 expressions in renal papilla were detected by immunohistochemical staining. All photographs were shown with a 400-fold magnification. Positive expression was indicated by arrows.

There was no significant difference in the expression of AQP-1 between telmisartan group and control group, but the expression of AQP-1 was significantly decreased in the aliskiren group compared with the control group (Figure 3(a)), indicating that the downregulation of AQP-1 expression was achieved in the renal papilla by inhibiting renin activity rather than blocking AT_1_ receptor.

The renin expressions in kidney were decrease through additional supplementation of renin into the central nervous system in 1*α*(OH)ase knockout mice (Figure 3(b)). AQP-1 expressions in renal papilla were down regulated by injected renin into lateral ventricle in 1*α*(OH)ase knockout mice as shown in Figure 3(c).

The expressions of AQP-1 in renal papilla of mice in pari group and aliskiren group were significantly lower than that in control group, while the expressions of AQP-1 were almost undetectable in pari + aliskiren group (Figure 3(d)), suggesting that Vitamin D and inhibiting renin activity have synergistic effects on downregulation of AQP-1expression.

### 3.4. Vitamin D and Inhibiting Renin Activity Have Synergistic Effects on Upregulation of AQP-4 Expression in Renal Proximal Tubules

**Figure 4:**
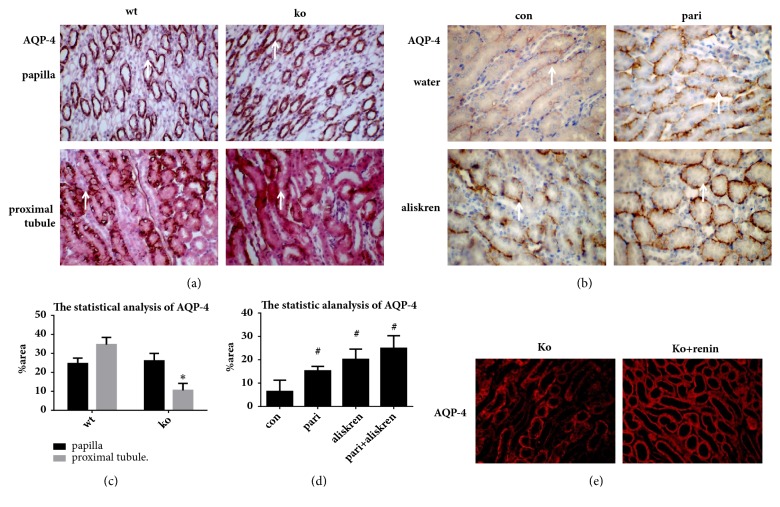
The synergistic effects of Vitamin D and inhibiting renin activity on upregulation of AQP-4 expression. (a) Immunohistochemical stain in kidney by AQP-4 antibody. (b) Immunohistochemical stain in renal proximal tubule by AQP-4 antibody. (c, d) The statistic analysis of (a) and (b) by Image J. (e) AQP-4 expressions in renal proximal tubule were detected by immunofluorescence staining. All photographs were shown with a 400-fold magnification. Positive expression was indicated by arrows. *∗* Compared with wt group, P<0.05. # Compared with wt group, P<0.05.

Although there were no differences in the papilla, the expressions of AQP-4 in the renal proximal tubules were significantly decreased in 1*α*(OH)ase knockout mice compared with the wild type (Figure 4(a)).

The AQP-4 expressions in renal proximal tubules were increased obviously in aliskiren group compared with control group (Figure 4(b)), suggesting that effects on AQP-4 were achieved by suppressing renin activity. Both Vitamin D and aliskiren upregulated AQP-4; however the expressions of AQP-4 were the most in combined group, suggesting that Vitamin D signaling pathway works synergistically with aliskiren to upregulate the expressions of AQP-4 in proximal tubules (Figure 4(b)). In addition, AQP-4 was high expressed in renal proximal tubule in Ko+renin group, in which the expressions of renin were decreased in kidney compared with ko group (Figure 3(b)).

Our results may indicate that Vitamin D works with inhibiting renin synergistically to upregulate of AQP-4 expressions in renal proximal tubules.

## 4. Discussion

In our early studies, we found that mouse with deletion of VDR gene had phenotype of polyuria and polydipsia [5]. Similar to VDR knockout mice, 1*α*(OH)ase knockout mouse is also characterized of increasing blood pressure and activation of the renin/angiotensin system due to lacking of 1,25-dihydroxyvitamin D_3_ [14]. In this study we found that 1*α*(OH)ase knockout mouse also developed polyuria, implying that Vitamin D may regulate production and excretion of urine in kidney.

Considering the role of AQPs in water transport, we first explored the expressions of AQP-1 and AQP-4 in knockout compared with wild-type mice. Interestingly, although there were no differences between the two type mice in renal proximal tubule, AQP-1 was highly expressed in renal papilla in 1*α*(OH)ase knockout mice compared with wild type (Figure 1). Paricalcitol (19-nor-1,25-dihydroxyvitamin D_2_) is an activated analog of Vitamin D to exert biological activities by recognizing VDR to further regulate target gene [15]. Its trophic activity appears to be highest of potency and efficacy at the doses used in our experimental model. In this study, the expressions of AQP-1 in mouse renal papilla were evident decreased by treating with paricalcitol. That was supposed that AQP-1 expressions could be regulated in renal papilla by activated Vitamin D or analog.

Schnermann's study shown that AQP-1 deletion in mouse resulted in decreasing transepithelial proximal tubule water permeability and defective fluid absorption [16]. Since urine is concentrated and accumulated in renal papilla before excreted, high expressions of AQP-1 were found in papilla instead of proximal tubule in1*α*(OH)ase knockout mice in our present study, and we infer that the high expression of AQP-1 in renal papillae may be responsible for the increase of water permeability in 1 alpha (OH) enzyme knockout mice.

Park's recently research showed that AQP-1 was obviously increased by giving Vitamin D analogue to the acute renal injury rats model induced by gentamicin [17], which was seem to be opposite of our results. In our study, we focused on the physiological conditions of mice, while Park's was based on acute renal injury of rats. In their study, AQP-1 was used as a bio-marker to evaluate the damage of kidney. The expression of AQP-1 was negatively correlated with renal injury. Vitamin D exerted a protective effect in gentamicin-induced renal injury by inhibiting renal inflammation and fibrosis. In rats with acute kidney injury, administration of vitamin D analogues can alleviate kidney injury, possibly by increasing the expression of AQP-1.

AQP-4 is mainly distributed in brain, kidneys, lungs, stomach, and small intestine and plays a key role in water homeostasis of brain edema [18, 19]. Solenov's study showed that the water permeability of primary astrocytes decreased by seven times in AQP-4 knockout mice [13]. And in Verkman's study, fourfold reduction of water permeability in inner medullary collecting duct was observed in AQP-4 knockout mice [20]. Our data showed that the expression of AQP-4 in 1 alpha (OH) enzyme knockout mice was significantly lower than that in wild type mice. Therefore, we infer that the polyuria phenotype of 1alpha (OH) enzyme knockout mice can be partially attributed to reduced water resorption through decreased AQP-4 expression in renal tubules.

Lütken confirmed that AT_1_ receptor inhibitor cambishtan could reduce the expression of AQP-2 in kidneys of mice with heart failure [21], suggesting that RAS may play a role in regulating the expression of AQP-2 in kidneys of mice. Considering that 1,25-dihydroxyvitamin D_3_ is a negative endocrine regulator of the renin-angiotensin system [22, 23], we hypothesize that Vitamin D may regulate AQP-1 and AQP-4 through blocking RAS. Telmisartan is an angiotensin II type I receptor blocker and aliskiren directly inhibits renin by inhibiting renin activity. Telmisartan and aliskiren were used to block the RAS pathway respectively in our study. Unexpectedly, there was no difference in the expression of AQP-1 in renal papilla between telmisartan group and control group, suggesting that blocking AT_1_ receptor would not affect the expression of AQP-1 in renal papilla. Compared with the control group, the expression of AQP-1 decreased in the renal papillae of the aliskren group, indicating that the expression of AQP-1 was downregulated by the inhibition of renin activity in the renal papilla. Renin supplementation in the central nervous system can reduce the negative regulation of renin in the renal papilla, thereby reducing the expression of AQP-1 (Figure 3). These results confirmed that Vitamin D signaling pathway may downregulate AQP-1 expressions in renal papilla by synergistic effects of inhibiting renin activity instead of classical RAS pathway.

In addition, as shown in Figure 4, the synergistic effect of paricalcitol and aliskiren regulates the expression of AQP-4 in proximal tubules.

Overall, Vitamin D and renin inhibitors have synergistic effects in regulating water channels in mice kidneys.

## Data Availability

The data used to support the findings of this study are available from the corresponding author upon request.
